# Genetic Ablation of Neural Progenitor Cells Impairs Acquisition of Trace Eyeblink Conditioning

**DOI:** 10.1523/ENEURO.0251-19.2019

**Published:** 2019-10-02

**Authors:** Lisa N. Miller, Craig Weiss, John F. Disterhoft

**Affiliations:** Department of Physiology, Feinberg School of Medicine, Northwestern University, Chicago, Illinois 60611

**Keywords:** dentate gyrus, learning, memory, neurogenesis, trace eyeblink conditioning

## Abstract

Adult-born neurons are believed to play a role in memory formation by providing enhanced plasticity to the hippocampus. Past studies have demonstrated that reduction of neurogenesis impairs associative learning, but these experiments used irradiation or neurotoxic substances, which may have had unintended off-target effects. Therefore, to investigate the role of these adult-born neurons more precisely, we used nestin-HSV-TK transgenic mice (Nes-TK) to selectively ablate newborn neurons. Nes-TK mice were fed a chow infused with valganciclovir to induce the ablation of neural progenitor cells. After being on this diet for 4 weeks, mice were trained on trace eyeblink conditioning, a hippocampus-dependent temporal associative memory task. Following the completion of training, brain sections from these animals were stained for doublecortin, a marker for immature neurons, to quantify levels of neurogenesis. We found that male transgenic mice on valganciclovir had significantly decreased amounts of doublecortin relative to male control animals, indicating a successful reduction in levels of neurogenesis. In conjunction with this reduction in neurogenesis, the male transgenic mice on valganciclovir learned at a significantly slower rate than male control mice. The female Nes-TK mice on valganciclovir showed no significant decrease in neurogenesis and no behavioral impairment relative to female control mice. Ultimately, the results are consistent with, and expand upon, prior studies that demonstrated that adult-born neurons are involved in the formation of associative memories. This study also provides a foundation to continue to explore the physiological role of newborn neurons with *in vivo* recordings during behavioral training.

## Significance Statement

Newborn neurons in the adult brain have been shown to be involved in associative learning, but many prior studies illustrating this point used neurotoxins or irradiation to ablate newborn neurons, which may have had unintended off-target effects. Therefore, we used a transgenic mouse model to eliminate adult-born neurons in a more controlled, precise manner. Ultimately, we demonstrate that the reduction of neurogenesis leads to an impairment in learning in males, and that levels of neurogenesis are associated with the rate of learning and overall performance on trace eyeblink conditioning.

## Introduction

Neurogenesis in the adult brain occurs in the dentate gyrus (DG) and produces new neurons that mature into granule cells and integrate into existing circuitry (Altman and Das, 1965; Dayer et al., 2003). These highly excitable neural progenitor cells are believed to play a role in memory formation by providing enhanced plasticity to the hippocampus (Snyder et al., 2001; Mongiat et al., 2009; Suter et al., 2019). Indeed, there are some studies that have found that reducing the number of newborn neurons impairs memory acquisition on different associative memory tasks. Specifically, the ablation of newborn neurons through systemic administration of an antimitotic agent prevented male rats from learning trace eyeblink conditioning (tEBC; Shors et al., 2001). Additionally, the elimination of adult-born neurons in male rats through fractionated irradiation led to an impairment in the hippocampal-dependent place recognition test, but had no effect on the hippocampal-independent object recognition task (M’Harzi et al., 1991; Steckler et al., 1998; Madsen et al., 2003). However, there have also been conflicting results depending on the species and methodology used to reduce neurogenesis. Snyder et al. (2005) observed that eliminating newborn neurons through irradiation did not impact acquisition on the hippocampal-dependent Morris water maze (MWM), but did impair long-term memory on this task. However, a study in male mice failed to see an impact of ablating newborn neurons on MWM performance (Saxe et al., 2006). Furthermore, while systemic administration of an antimitotic agent reduced freezing in male rats during trace, but not contextual, fear conditioning (Shors et al., 2002), the elimination of newborn neurons through irradiation and genetic manipulations in male mice led to a reduction in freezing behavior during contextual, but not trace, fear conditioning (Saxe et al., 2006). These inconsistencies therefore warrant further investigation into the involvement of adult-born neurons in associative learning.

A limitation of these past studies is that the large majority of them made use of irradiation or neurotoxic substances that may have had unintended off-target effects. For example, the antimitotic agent methylazoxymethanol acetate (MAM) used in some studies has been shown to impact the overall health of an animal and to induce hypoactivity (Dupret et al., 2005). With newer genetic techniques, however, we can investigate whether adult-born neurons are necessary for the acquisition of associative learning with greater precision and fewer potential confounds. Specifically, we used nestin-HSV-TK transgenic mice (Nes-TK) to selectively reduce the number of newborn neurons to investigate the role of these neural progenitor cells (Yu et al., 2008). These mice express a modified herpes simplex virus thymidine kinase driven by a nestin promoter and its second intron regulatory element, which allows for temporally regulated induced ablation of dividing neural progenitors through systemic administration of ganciclovir or its prodrug valganciclovir (Val; Yu et al., 2008; Mustroph et al., 2015).

To study whether adult-born neurons are necessary for learning, we trained animals on tEBC, a hippocampal-dependent temporal associative memory task in which an otherwise neutral conditioned stimulus (CS) is paired with an aversive unconditioned stimulus (US) that causes a reflexive eyeblink response. The two stimuli are separated in time by a stimulus-free trace interval, and animals learn to associate the two stimuli over many trials. On learning this association, animals start to close their eye during the trace interval in anticipation of the US, which is known as the conditioned response (CR). The advantages of tEBC are that it takes many trials for animals to learn, which allows for comparisons of the rate of learning, as well as the ability to look at changes in cellular activity over the course of learning through *in vivo* recording methods.

The goal of this study was to address whether genetic ablation of neural progenitor cells affects the acquisition of tEBC, using both male and female mice. Ultimately, we found that a reduction in the number of newborn neurons impairs the acquisition of this hippocampus-dependent temporal learning task, and that levels of neurogenesis are correlated with overall performance and rate of learning in male mice.

## Materials and Methods

### Animals

Animal care procedures were conducted in accordance with National Institutes of Health guidelines and as approved by the Northwestern University Institutional Animal Care and Use Committee. The Nes-TK transgenic mouse line (stock #029671, The Jackson Laboratory; RRID:IMSR_JAX:029671) was originally developed in the laboratory of S.G. Kernie (Columbia University, New York, NY; Yu et al., 2008). Mice were bred in a Northwestern University animal facility, and the genotype of each animal was determined by a tail snip sample sent to Transnetyx. Both male and female mice were used in this study, and the estrous cycle was not monitored (Prendergast et al., 2014; Fritz et al., 2017).

Four weeks before behavioral training, at ∼8–14 weeks of age, mice were singly housed and provided *ad libitum* access to either regular chow or chow infused with valganciclovir (1350 mg/kg; Custom Animal Diets), a valine ester prodrug of ganciclovir. This schedule was based on the findings from the study by Shors et al. (2001) that demonstrated that newborn neurons in rats are ∼1–2 weeks old when they become involved in learning tEBC. However, there is a 1–2 week delay in the maturation of young granule cells in mice compared with rats (Snyder et al., 2009), which is why mice were started on their given diet 4 weeks before tEBC. Assigned diets were maintained until the animals were killed. The experimental group consisted of Nes-TK mice eating Val-chow, and the three control groups included Nes-TK mice eating regular chow, wild-type (WT) mice eating Val-chow, and WT mice eating regular chow. These control groups were used to investigate whether the drug or genotype alone would have an effect on learning. Ultimately, after there was no observed difference in learning among the three groups, they were combined into one control group to avoid using animals unnecessarily. Val-chow was weighed weekly to monitor food intake to calculate average Val dosage.

### Trace eyeblink conditioning

Two weeks before the start of behavioral training, mice underwent headbolt implantation surgery, during which subdermal wires were placed around the orbicularis oculi muscle to measure eyeblink response via electromyography (EMG) activity. After 1 week of recovery, mice were handled for 5 min/d for 3 d and then habituated to the training chamber for 2 d, for ∼45-60 min/d. Finally, animals were trained two at a time on tEBC for 10 d.

During tEBC, mice were head-fixed atop a freely rotating cylinder (Heiney et al., 2014; Lin et al., 2016). Each session was composed of 40 trials, each consisting of a 250 ms whisker displacement (i.e., CS; ±50 µm at 62 Hz, delivered by a comb attached to a piezo actuator) paired with a 30 ms air puff to the cornea (i.e., US; 3.5 psi, delivered by a blunted 16 gauge needle pointed at the eye). Presenting the CS and US to different sensory modalities prompts learning that requires the integration of sensory information at higher-level cortical structures. This is in contrast to other trace-conditioning paradigms where the CS and US are both applied to the whisker pad (Troncoso et al., 2004), which may be mediated by motor neurons or the brainstem. The two stimuli were separated by a 250 ms stimulus-free trace interval, and the mean intertrial interval was 45 s (range, 30–60 s). White noise (78–80 dB) was played for the duration of each session. Air pressure and vibration intensity were calibrated between each set of animals using a manometer (Thermo Fisher Scientific) and a displacement sensor (optoNCDT 1320, Micro-Epsilon), respectively. The displacement sensor also provided real-time confirmation that the whisker vibration was delivered during each trial. Custom LabView software was used to control stimuli presentation, data collection, and data analysis. CRs were defined as EMG activity during the 200 ms before US presentation that was >4 SDs above baseline for >15 ms, with the baseline being defined as the 250 ms before CS onset. An animal was considered to reach the learning criterion when it demonstrated CRs for at least 60% of trials within a session.

### Immunohistochemistry

Two weeks after the completion of training, mice underwent an intracardial perfusion with 0.1 m PBS followed by 4% paraformaldehyde (PFA). The brain was removed and stored in 4% PFA overnight at 4°C. The following day the brains were rinsed with PBS and stored in 30% sucrose in PBS for cryoprotection. Brains were sliced on a freezing microtome into 40 µm coronal sections, and a one in six series of the dorsal hippocampus (approximately −1.5 to −2.7 mm posterior to bregma; a total of six sections) was selected from each brain for immunofluorescent staining.

Immunofluorescent staining was performed as per the protocol from the laboratory of J.S. Rhodes (University of Illinois at Urbana–Champaign, Urbana–Champaign, IL; Mustroph et al., 2015). Free-floating sections were first washed with Tris-buffered saline (TBS). To denature DNA, the sections were then treated with a solution of 50% deionized formamide and 2× saline-sodium citrate for 2 h at 65°C, washed in 2× saline-sodium citrate for 15 min, treated with 2 m hydrochloric acid for 30 min at 37°C, and washed with 0.1 m borate buffer for 10 min. After a rinse in TBS, sections were blocked in a solution of TBS with 3% normal goat serum and 0.3% Triton X-100 (TBS-X) for 30 min before incubating in a primary antibody dilution in TBS-X at 4°C for 48 h. Sections were then washed with TBS, blocked in TBS-X for 30 min, and incubated in a secondary antibody dilution in TBS-X for 3 h. Primary antibodies used were rabbit anti-doublecortin (DCX; 1:250; Abcam; RRID:AB_732011) and mouse anti-neuronal-specific nuclear protein (NeuN; 1:500; Abcam; RRID:AB_10711040). Secondary antibodies used were Invitrogen donkey anti-rabbit and goat anti-mouse (all 1:250; Thermo Fisher Scientific; RRID:AB_141637 and RRID:AB_2535804) and were conjugated to Alexa Fluor 594 and 647, respectively.

### Quantification and statistical analysis

All sections were imaged on a confocal microscope with a 20× objective to visualize the entire granule cell layer of dentate gyrus in each of the six sections per animal. A *z*-stack of images was produced to encompass the complete thickness of the granule cell layer. Using NIS Elements software (Nikon), a maximum intensity projection was created in the *z*-plane to use for cell counting. The area of the granule cell layer and the number of DCX^+^ cells within the granule cell layer were calculated using a custom analysis in NIS Elements. The volume of each dentate gyrus section was calculated by multiplying this calculated area with the section thickness so that the average number of DCX^+^ cells could be expressed per cubic micrometer of dentate gyrus.

All statistical analyses were performed with StatView, with *p* < 0.05 considered to be statistically significant. Repeated-measures ANOVA was used to compare learning curves, with different groups as the independent variable and training day as the repeated measure. Unpaired *t* tests were used to compare groups on each day of training, as well as the number of DCX^+^ cells between the experimental and the combined control groups. Pearson’s correlation coefficient was used to examine the relationship between the number of DCX^+^ cells and various measures of learning. Outliers were removed if they exceeded 2 SDs beyond the mean; based on this criterion, one male animal was excluded from the experimental group. Data are expressed as the mean ± SEM. A complete list of statistical tests and results can be found in Table 1.

**Table 1.** Statistics table

Description	Type of test	Sample size	Statistical data
Figure 1: Comparison of learning curves			
Male control groups	RM ANOVA	WT/Reg: *n* = 3 WT/Val: *n* = 3 Nes-TK/Reg: *n* = 5	Group: *F* = 3.84 *p* = 0.0677
Female control groups	RM ANOVA	WT/Reg: *n* = 3 WT/Val: *n* = 1 Nes-TK/Reg: *n* = 2	Group: *F* = 0.51 *p* = 0.6454
Male vs female controls	RM ANOVA	Male: *n* = 11 Female: *n* = 6	Group: *F* = 0.63 *p* = 0.6346
Male; Con vs Exp	RM ANOVA	Con: *n* = 11 Exp: *n* = 13	Group: *F* = 15.2 *p* = 0.0008 Interaction: *F* = 1.837 *p* = 0.0487
Male; Con vs Exp All sessions	Unpaired *t* tests	Con: *n* = 11 Exp: *n* = 13	H1: *p* = 0.06 H2: *p* = 0.24 T1: *p* = 0.022 T2: *p* = 0.0071 T3: *p* = 0.0011 T4: *p* = 0.029 T5: *p* = 0.018 T6: *p* = 0.0017 T7: *p* = 0.0087 T8: *p* = 0.0045 T9: *p* = 0.034 T10: *p* = 0.062
Female; Con vs Exp	RM ANOVA	Con: *n* = 6 Exp: *n* = 5	Group: *F* = 0.89 *p* = 0.3696
Figure 2: Comparing number of DCX^+^ cells			
Male; Con vs Exp	Unpaired *t* test	Con: *n* = 11 Exp: *n* = 13	*p* = 0.0011
Female; Con vs Exp	Unpaired *t* test	Con: *n* = 6 Exp: *n* = 5	*p* = 0.0947
Figure 3: Correlation between learning and number of DCX^+^ cells			
Male; DCX vs average CRs	Pearson’s correlation	*n* = 24	*r* = 0.574 *p* = 0.0027
Male; DCX vs trials to 6 CRs of 10 trials	Pearson’s correlation	*n* = 24	*r* = −0.519 *p* = 0.0121
Female; DCX vs average CRs	Pearson’s correlation	*n* = 11	*r* = −0.025 *p* = 0.943
Female; DCX vs trials to 6 CRs of 10 trials	Pearson’s correlation	*n* = 11	*r* = 0.102 *p* = 0.77

All datasets are assumed to have normal distribution. Con, control; Exp, experimental; Reg, regular chow; Val, valganciclovir chow; RM, repeated measures.

## Results

To ablate neural progenitor cells, Nes-TK mice were placed on a Val-infused diet 4 weeks before behavioral training. These mice were considered the experimental group, and both male and female mice exceeded the desired Val dosage of 200 mg/kg/d (215 ± 8.2 and 235 ± 9.3 mg/kd/d, respectively; Yu et al., 2008). The control groups consisted of Nes-TK mice eating regular chow, WT mice eating Val-chow, and WT mice eating regular chow.

**Figure 1.**
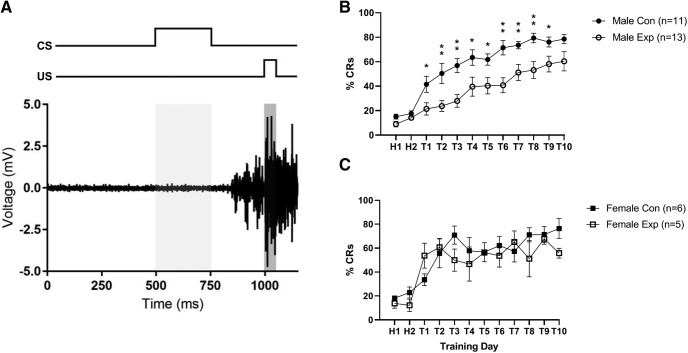
Trace eyeblink conditioning in mice. ***A***, EMG activity from an animal trained on tEBC (bottom trace), depicting a well timed CR. The timing of the CS (whisker vibration) and US (air puff) presentation are shown at the top of the panel. ***B***, ***C***, Learning curves for the male (***B***) and female (***C***) control and experimental groups. Average percentage CRs are shown for each day, where “H” refers to days of habituation and “T” refers to days of training. Error bars represent the SEM. *Post hoc t* tests were used to test statistical differences for each day of training (^*^p < 0.05; ^**^p < 0.01).

All mice were trained on tEBC for 10 d, with 40 trials per session. The EMG activity of the mice was monitored for CRs preceding US presentation, and an animal was considered to have reached learning criterion when it showed CRs for at least 60% of trials within any session. An example EMG trace of a well timed CR is shown in Figure 1*A*. There was no significant difference in learning among the three types of male controls (*F*_(2,8)_ = 3.84, *p* = 0.0677), so they were combined into one control group, which is depicted in Figure 1*B*. The same was done for the female control group (*F*_(2,3)_ = 0.51, *p* = 0.6454), shown in Figure 1*C*. Additionally, there was no significant difference in learning between the male and female control groups (*F*_(1,15)_ = 0.63, *p* = 0.6346). These two groups learned at approximately the same rate, reaching learning criterion on days 4 and 3 of training, respectively (Fig. 1*B*,*C*).

The male experimental group learned at a significantly slower rate than the male control animals (*F*_(1,22)_ = 15.2, *p* = 0.0008), reaching learning criterion on day 10 of training (Fig. 1*B*). Additionally, there was a significant interaction between group and training day for male animals (*F*_(11,242)_ = 1.837, *p* = 0.0487). Unpaired *t* tests revealed that there was a significant difference between the experimental and control groups starting on the first day of training (*p* = 0.022), but this difference was no longer present by the final day of training (*p* = 0.062). Training days 2 through 9 were also significantly different (*p* < 0.05), and a complete list of *p* values can be found in Table 1. Female Nes-TK mice on the Val diet showed no difference in learning relative to the female control group (*F*_(1,9)_ = 0.89, *p* = 0.3696; Fig. 1*C*).

**Figure 2.**
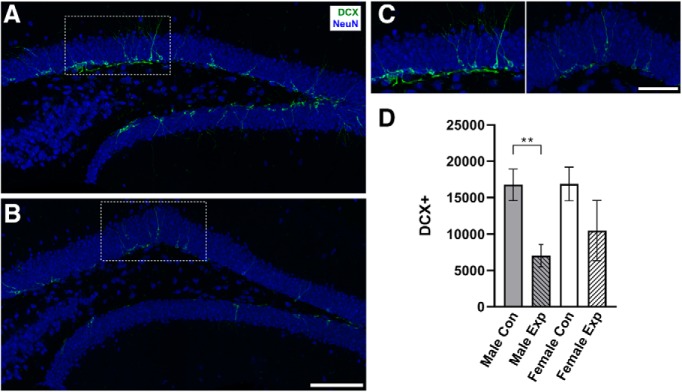
Measuring neurogenesis in the adult brain. ***A***, ***B***, Sample images of DCX expression in DG in sections from a male control animal (***A***) and a male experimental animal (***B***). ***C***, Zoomed in views of DCX^+^ cells from ***A*** and ***B*** (left and right, respectively). ***D***, Quantification of the number of DCX^+^ cells within the granule cell layer, expressed as the number of cells per cubic micrometer. The male experimental group (*n* = 13) showed a significant decrease relative to the male control group (*n* = 11; ^**^p < 0.01). There was no significant difference between the female experimental group (*n* = 5) and the female control group (*n* = 6; *p* > 0.05). Error bars represent the SEM. Scale bars: ***B***, 100 µm; ***C***, 50 µm.

Two weeks after the completion of training on tEBC, the number of DCX^+^ cells in DG was quantified to validate the effect of valganciclovir on neurogenesis in Nes-TK mice (Fig. 2). DCX is a microtubule-associated protein expressed by immature neurons that is used as a marker for neurogenesis (Brown et al., 2003; Couillard-Despres et al., 2005). The male experimental mice had significantly fewer DCX^+^ cells relative to the male control group (7054 ± 1529 and 16,754 ± 2165 cells/µm^3^, respectively), showing a 58% decrease in the number of immature neurons (*p* = 0.0011). Female experimental mice, however, only showed a nonsignificant 38% decrease relative to the female control group (10,487 ± 4120 and 16,886 ± 2304 cells/µm^3^, respectively; *p* = 0.0947; Fig. 2*D*).

**Figure 3.**
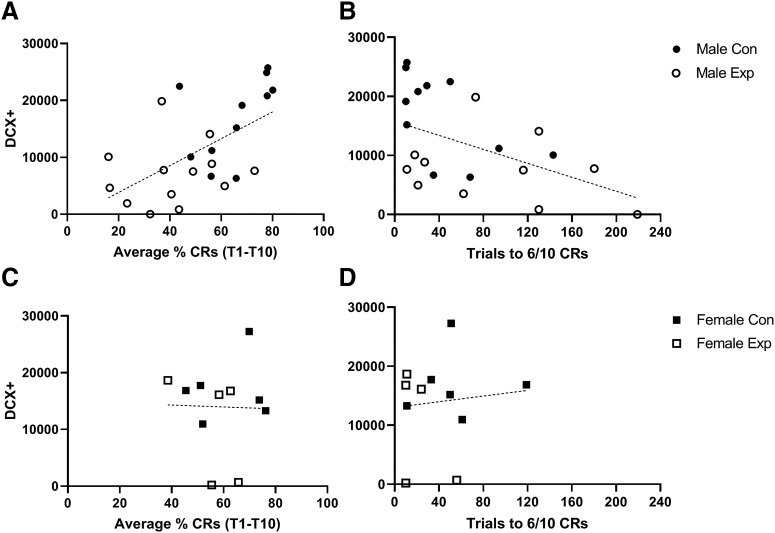
The amount of neurogenesis is correlated with learning in male mice. ***A***, ***C***, The number of DCX^+^ cells per micrometer of DG is positively correlated with the average percentage of CRs across all 10 d of training for males (***A***; *r* = 0.574, *p* = 0.0027), but not females (***C***; *r* = −0.025, *p* = 0.943). ***B***, ***D***, The number of DCX^+^ cells per cubic micrometer of DG is negatively correlated with the number of trials it took to show six CRs within a sliding block of 10 trials for males (***B***; *r* = −0.519, *p* = 0.0121), but not for females (***D***; *r* = 0.102, *p* = 0.77).

A significant positive correlation was observed between the number of DCX^+^ cells and the average percentage of CRs across all 10 d of training for male animals (*r* = 0.574, *p* = 0.0027), indicating that male animals that had higher levels of neurogenesis performed better overall (Fig. 3*A*). Similarly, there was a significant negative correlation between the number of DCX^+^ cells and the number of trials it took for a mouse to display six CRs within a sliding 10-trial block for male animals (*r* = −0.519, *p* = 0.0121), suggesting that male animals that had more newborn neurons learned at a faster rate (Fig. 3*B*). Female animals, however, showed no significant correlation between the number of DCX^+^ cells and either of these measures of learning (*r* = −0.025, *p* = 0.943; and *r* = 0.102, *p* = 0.77, respectively; Fig. 3*C*,*D*), although the Val diet may not have been as effective in our female mice as it was in our male mice.

## Discussion

This study used genetic ablation of neural progenitor cells to explore whether adult neurogenesis is necessary for associative learning in mice. Ultimately, we found that decreasing neurogenesis led to an impairment in the acquisition of tEBC. Male Nes-TK mice on valganciclovir showed a nearly 60% reduction in the number of DCX^+^ cells and learned at a significantly slower rate than male control animals. Thus, newborn neurons are indeed involved in temporal associative learning, a finding that is in accordance with a previous study that used MAM to diminish the number of adult-born neurons in rats (Shors et al., 2001). Following this neurotoxic ablation of newborn neurons, Shors et al. (2001) observed a significant impairment in the ability of rats to learn tEBC. This previous study only eliminated newborn neurons born 1–2 weeks before training, while our study inhibited neurogenesis continuously before and during tEBC. However, our animals were able to eventually reach learning criterion by the end of training, unlike the results reported in the previous study where rats injected with MAM failed to reach the criterion. Our findings indicate that adult-born neurons contribute to learning this temporal association but are not the only dentate gyrus neurons that contribute. These other neurons are likely other types of dentate gyrus neurons or existing mature granule cells, neither of which were affected by our manipulation. The fact that the rats injected with MAM were unable to reach the learning criterion was likely due to nonspecific side effects of MAM, or possibly due to a species difference. It should be stressed that we observed impairment in learning starting on the very first training day while the previous study (Shors et al., 2001) did not, which further emphasizes the important role of adult-born neurons in learning.

In addition to an impairment in the rate of learning, we found that neurogenesis was correlated with various measures of learning in male mice. We observed a positive correlation between the number of DCX^+^ cells and the average percentage of CRs across all 10 d of training, which suggests that male animals with more newborn neurons tended to perform better overall. This result is consistent with previous reports that demonstrated the same positive correlation between the number of newborn neurons and the average percentage CRs in rats trained on tEBC (Curlik and Shors, 2011). We also observed a significant negative correlation between the number of DCX^+^ cells and the number of trials it took for male animals to display six CRs within a moving block of 10 trials, indicating that male animals that had higher levels of neurogenesis learned faster. This is the opposite of the correlation reported by Curlik and Shors (2011), who found that rats that took longer to learn tEBC showed a higher retention of neurons born before the beginning of training. These findings are not mutually exclusive, as Curlik and Shors (2011) injected bromodeoxyuridine before behavioral training to examine the survival of newborn neurons, while our study compared the overall production of adult-born neurons. Thus, while slower learning may be associated with increased survival of new neurons, our data suggest that animals with higher levels of neurogenesis learn at a faster rate. Interestingly, we observed no significant correlations between the number of DCX^+^ cells and either measure of learning for female animals. This would suggest that females make use of alternative cell types or mechanisms to acquire tEBC and/or that females are less affected by the Val diet. Prior studies have demonstrated that not only do females use different strategies for spatial navigation, but that neurogenesis is differentially correlated with performance on a radial arm maze task depending on what strategy is used (Yagi et al., 2016).

Interestingly, we observed no behavioral effect in the Nes-TK female mice on valganciclovir. This is likely due to the fact that there was no significant reduction in neurogenesis relative to the female control group. It is possible that this manipulation was not effective in the females due to some degree of genetic drift during colony creation or maintenance, as other studies have used female Nes-TK mice with clear success (Hollands et al., 2017). This could also explain why we did not see as large a decrease in neurogenesis in the male animals as was observed in the initial study that used this transgenic line (Yu et al., 2008). Additionally, the male and female control groups learned at the same rate, unlike previous reports that found that female rats learned faster than males (Dalla et al., 2009). A possible reason for this disparity could be the difference in species, as the current study used mice while Dalla et al. (2009) used rats. Another possibility is the difference in the behavioral task, as the CS and US in the previous study were white noise and a shock to the eyelid, respectively, with a trace period of 500 ms (Dalla et al., 2009), while we used a 250 ms trace interval in the current study, with a whisker vibration CS and air puff US. With a longer trace period, and more trials needed to reach the learning criterion, the previous behavioral paradigm may have been more difficult to learn than the paradigm used in the current study. Therefore, it is also possible that we could observe a difference in the rate of learning between male and female mice if we used a longer trace interval. Regardless of these differences, our results clearly show in male mice that reduced neurogenesis in the dentate gyrus impairs the rate of hippocampal-dependent eyeblink conditioning.

Recent work by Suter et al. (2019) discovered cells within dentate gyrus that showed changes in the firing rate that started at CS onset and persisted through the trace period during tEBC. This finding suggests that there are cells within DG that are bridging the temporal gap between stimuli, which would play a vital role in associative learning. Because the cells that showed this persistent firing were highly excitable, the cell type was hypothesized to be either newborn neurons or mossy cells, as both are more excitable than granule cells (Mongiat et al., 2009; GoodSmith et al., 2017; Senzai and Buzsáki, 2017). Additionally, recent work by Madroñal et al. (2016) has demonstrated that while mature granule cells within DG are not involved in the retrieval of tEBC memory, transient inhibition of these cells led to a rapid, transient decrease in conditioned responses. This suggests that mature dentate granule cells are involved in the maintenance of associative memory. In the present study, we have demonstrated that adult-born neurons are indeed involved in acquiring tEBC, which provides a foundation to further explore exactly how newborn neurons and other cell types in dentate gyrus contribute to associative learning through *in vivo* recording techniques during behavioral training.

## Synthesis

Reviewing Editor: Mark Laubach, American University Decisions are customarily a result of the Reviewing Editor and the peer reviewers coming together and discussing their recommendations until a consensus is reached. When revisions are invited, a fact-based synthesis statement explaining their decision and outlining what is needed to prepare a revision will be listed below. The following reviewer(s) agreed to reveal their identity: Kaori Takehara-Nishiuchi, Agnès Gruart.

The reviewers and I felt that you should address several minor issues raised in the reviews, and most importantly that you should revise the text extensively to more clearly state the novelty of the findings. A major concern was that the results were incremental, and the novelty of the study in comparison to previous publications on the topic was not clear to all. Please be sure to address this issue before you resubmit the manuscript.

REVIEWER 1

The authors investigated the necessity of adult-born neurons for associative learning. By using an inducible transgenic mouse model that allows for genetically ablating adult-born neurons at a specific time, they showed male mice with suppressed neurogenesis were impaired in memory acquisition in trace eyeblink conditioning. In contrast, the same manipulation applied to female mice did not significantly impair memory acquisition. Consistent with the sex difference in the effect on memory, the manipulation significantly reduced the number of adult-born neurons only in the male mice. Moreover, the task performance was positively correlated with the number of adult-born neurons in dentate gyrus. These findings confirm the relevance of neurogenesis in hippocampus-dependent associative learning and set the stage for investigating their physiological role in future in vivo recording experiments.

The manuscript is clearly written. The results and interpretations are straightforward. Their novelty and significance, however, are not clear because the present findings appear to be a simple replication of the previous finding (Shors et al., 2001) with a different methodology. Nonetheless, below I list some suggestions for minor edits.

EDITOR: The issue raised by Reviewer 1 is the major issue that you should address in revising the manuscript, and in your rebuttal. Please be sure to provide an extensive comment about how your study goes beyond the Shors study (and the related papers that are cited) and how your methodology extends the current literature.

1) The experimental group received a valganciclovir-infused diet for four weeks prior to behavioral training. Please justify why this schedule was chosen.

EDITOR: Please address this issue in the revised manuscript.

2) I suggest that the authors discuss the difference in the effect on the number, maturation, and integration of new-born neurons between their genetic manipulation and the manipulation used in the previous study (Shorts et al., 2001). This will highlight exactly which aspect of neurogenesis (e.g., a presence of immature, new-born neurons or mature new-born neurons integrated into the existing network) is necessary for associative learning.

EDITOR: Please focus on this issue in the revised manuscript in the Intro and Discussion.

REVIEWER 2

In the present study it is reported that the genetic ablation of neural progenitor cells prevent the acquisition of trace eyeblink conditioning in male, but not in female, mice. Presently, there are contradictory results regarding neurogenesis in the dentate gyrus of young adult mice. But, as reported here, hippocampal neurogenesis plays a significant role in the acquisition of classical eyeblink conditioning in head-inmovilized mice. I have just a few comments and suggestions aimed to clarify some experimental procedures and to determine the limitations of the study.

- page 4, 3rd paragraph. Why authors designed three different control groups (composed of a rather small number of animals) if they added them together in the same group?

EDITOR: Please provide a rationale for this aspect of the study in the main text.

- Page 5. Why the CS was presented to the vibrissae and the CR was recorded in the eyelids? In this way, authors were recording a generalized and, obviously, small CR. At the least, authors should commented an early study in which the CR was recorded directly from vibrissal muscles (Troncoso et al., Learn Mem, 2004).

EDITOR: Please comment on the impact of using whisker stimuli to elicit eyelid movements in the main text, and also comment on how your measures might be different if EMG recordings were used as in the Troncoso study.

- Figure 1. Please indicate in B the significant differences collected for each single session. In the text is just mentioned that there was a significant diference for data collected for T1, but additional infomation is requested for the rest of training sessions. Same comment applies to results comented in page 8, 1st paragraph.

EDITOR: Please address these issues in Figure 1 and the text cited by the reviewer. The figure needs some indicators for significant differences between the control and experimental groups in panel B, following the text on the top of page 8.

- Figure 2. Neuron labelling is almost invisible in A and B. Please improve the quality of selected staining.

EDITOR: Please revise this figure. (I struggled to read the blue text, myself.)

- Figure 3. Data presented in this figure are rather surprising because there is not a clear separation from results collected from the control and the experimental group. Please, check.

EDITOR: The separation is not dramatic, and I do not understand why you removed the females from the plots. Please provide alternative plots with both sexes, and consider using all data for the Figure 3.

Discussion section. I imaging that the disappearance of newborn neurons in the dentate gyrus is a rather slow process. Interestingly enough, it has been reported recently that the transient inativation of dentate gyrus activity in behaving mice evokes a significant erase of classically conditioned eyelid responses (Madronal et al., Nature Commun, 2016). This study should be commented with regard to the putative role of dentate granule and/or mossy neurons in this type of associative learning.

EDITOR: Please include discussion of Madronal in the revised manuscript.

## Author Response

We are very thankful to the reviewers for their feedback and insight. We have revised the manuscript according to the reviewers' suggestions and a detailed response to all their comments can be found below, including references to which lines contain the revised text.

1. The reviewers requested that the text be revised to emphasize the novelty of the findings. We have modified the introduction and discussion sections to highlight how our findings are different from previous studies and how this paper adds to the current literature (lines 55-56, 58-61; 235-246). We emphasize that our study is based in mice instead of rats, and given that adult-born hippocampal neurons are more numerous, faster-maturing and more involved in behavior in rats than in mice (Snyder et al., 2009), we find it valuable to have been able to demonstrate this link between neurogenesis and temporal associative learning in mice. Additionally, we would like to highlight the fact that we included both sexes instead of only using male subjects as previously done, and that we report a difference in neurogenesis and learning rate between the sexes.

2. Given that mouse neurogenesis occurs more slowly than in rats (see previous comment), we began the valganciclovir diet four weeks prior to training, as described in the methods section (lines 98-102).

3. The reviewers suggested that we elaborate further on how the manipulations done in our study vary from the manipulations done in Shors et al. (2001), which we have done as elaborated on in Response 1.

4. We have added rationale for the purpose of each of the three separate control groups and why they were combined (lines 105-108).

5. The reviewers requested clarification on the reasoning for recording EMG from the eyelid and to comment on the differences of our behavior as compared to Troncoso et al. (2004). In our paradigm, the whisker vibration serves as an otherwise neutral stimulus and the eyeblink reflex is elicited by the airpuff directed to the cornea (the unconditioned stimulus). In this instance it is more appropriate to record from the eyelid, as opposed to the Troncoso study that used a whisker stimulus as the unconditioned stimulus and thus recorded from the whisker-pad. Troncoso et al. devised their paradigm to test the evolutionary consequences of conditioned reflex circuitry rather than to examine the mechanisms within the primary conditioning circuit per se. We have also added discussion on how our study differs from the Troncoso study in our methods section (lines 122-125).

6. We have added notation on Figure 1B to distinguish the significance between groups for each day (i.e. p<0.05 and p<0.01). Additionally, we have added all the corresponding p-values to the statistics table (Table 1) and referenced this in the text (lines 202-204).

7. As requested, we have adjusted Figure 2 so that the text is easier to read, as well as adding zoomed-in boxes to improve visibility/provide emphasis on the DCX+ neurons.

8. The reviewers asked for clarification on the results in Figure 3, as there is not a clear separation between results collected from the control and experimental groups. This plot is correct, however, as it is representative of the correlation coefficient that is reported in the body of the manuscript. The lack of clear separation can be attributed to differential responses to the diet or intrinsic variability among the mice (neurogenesis is inherently variable in nature), leading some control animals to have fewer DCX+ cells and some experimental animals to have more DCX+ cells. The main point of the correlation is that the degree of neurogenesis is related to the rate and degree of hippocampal dependent learning.

9. Per the reviewers' request, we have revised Figure 3 to also include the data from female subjects, and revised our text to reflect this change (lines 214-224, 262-268).

10. We have added discussion of the effect of transient inhibition of granule cells by Madronal et al. (2016) to the discussion section of the manuscript as requested (lines 295-299).
